# The Impact of Sarcopenia in Patients with Peritoneal Surface Disease

**DOI:** 10.3389/pore.2021.638857

**Published:** 2021-08-30

**Authors:** Aubrey Juris, Amanda Taylor-Gehman, Brianna Spencer, Eric Schaefer, Colette Pameijer

**Affiliations:** ^1^ Department of Surgery, College of Medicine, The Pennsylvania State University, Hershey, PA, United States; ^2^ Department of Family and Community Medicine, College of Medicine, The Pennsylvania State University, Hershey, PA, United States; ^3^ Department of Public Health Sciences, College of Medicine, The Pennsylvania State University, Hershey, PA, United States

**Keywords:** sarcopenia, muscle mass, cytoreduction, outcomes, intraperitoneal chemotherapy, peritoneal surface disease

## Abstract

Cytoreductive surgery and hyperthermic intraperitoneal chemotherapy is increasingly performed in patients with advanced cancer in the abdomen. This treatment prolongs survival for some patients but is known to have a substantial rate of complications. Choosing patients for this procedure can be difficult, and no clear guidelines exist. Muscle mass is a general measure of a patient’s wellness, meaning that patients with low muscle mass for their body weight tend to have more complications from treatment and overall do worse. We evaluated muscle mass prior to surgery in our Cytoreductive surgery/hyperthermic intraperitoneal chemotherapy population to assess how many patients have low muscle mass and the impact on outcomes, such as length of hospital stay, complications and survival. We find that about 25% of our patient population has low muscle mass, and low muscle mass is associated with a higher burden of cancer and shorter survival. We were able to evaluate muscle mass in a small number of patients after surgery, expecting to find decreased muscle mass in all the patients after a complex operation and long recovery. In fact, none of the patients had low muscle mass, including those who were low prior to surgery.

## Introduction

Cytoreductive surgery (CRS) and hyperthermic intraperitoneal chemotherapy (HIPEC) are increasingly performed for patients with peritoneal spread of gastrointestinal (GI) and gynecologic (GYN) malignancies and mesothelioma. Although this procedure has provided prolonged survival for patients, it is associated with a relatively high complication rate. Patient selection remains critical for success of this operation, but current selection criteria are either not oncologic (i.e. performance status, albumin level) or not modifiable (i.e. peritoneal cancer index (PCI), histology). We sought to determine an improved marker of patient outcome as well as a parameter for which intervention was possible.

Sarcopenia is degenerative loss of skeletal muscle mass. It is associated with decreased survival in the general population, and has also been associated with poor outcomes in patients with localized colorectal cancer, hepatocellular cancer, pancreatic cancer and women with ovarian cancer. Patients with sarcopenia have been found to have increased toxicity from intraperitoneal chemotherapy [1], and sarcopenic patients with colon cancer have poor outcomes with CRS-HIPEC [2]. We evaluated our HIPEC patient population to determine the frequency of sarcopenia, and assess for any association with length of stay (LOS), complications or post-HIPEC survival.

## Methods

**FIGURE 1:**
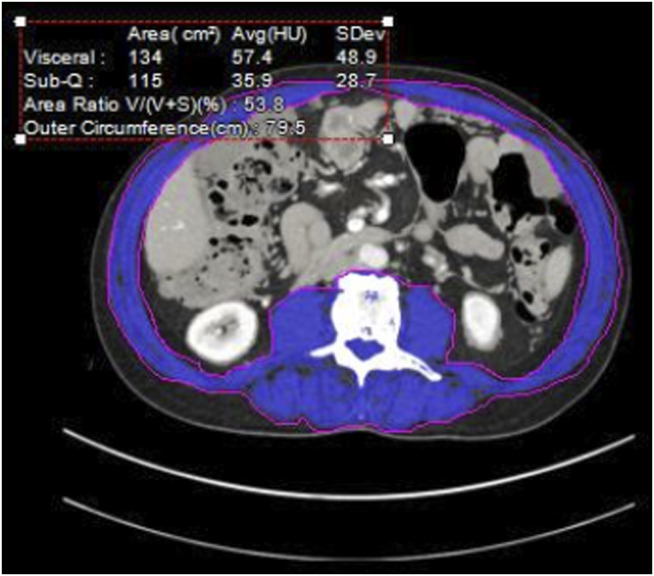
Cross sectional Abdominal CT at L3. Blue area—quantified muscle [3].

We performed a retrospective review of consecutive HIPEC patients at our institution from 11/2013–4/2018, with imaging available for analysis within 2 months prior to CRS/HIPEC. Multiple factors are considered when selecting patients for CRS/HIPEC including the origin and histology of the tumor, burden of disease, underlying medical problems and fitness for surgery, and extent and response to systemic agents. In uncertain cases we may perform diagnostic laparoscopy first to assess resectability. For patients who had 2 CRS/HIPEC procedures we utilized data from the first procedure only. Demographic variables including age, diagnosis, date of diagnosis, BMI, serum albumin, chemotherapy prior to HIPEC, postoperative complications, length of stay (LOS) and post-HIPEC survival were recorded. The burden of disease is routinely assessed for each patient with the Peritoneal *Cancer* Index (PCI), a standardized score [3] in which the largest tumor nodule in each of 13 different sections of the abdomen is measured. No disease is scored as 0, disease measuring up to 0.5 cm is scored as 1, up to 5 cm scored as two and >5 cm or confluent disease as 3. The PCI can range from 0–39. The Aquarius iNtuition imaging calculation software (TeraRecon, Durham, NC) [4] was used to assess the CT scans and calculate skeletal muscle mass as well as subcutaneous and visceral fat to evaluate for sarcopenia [5] (Figure 1). Sarcopenia was defined as skeletal muscle mass <52 cm^2^/m^2^ for normal or overweight men and <54 cm^2^/m^2^ for obese men, <38 cm^2^/m^2^ for normal or overweight women and <47 cm^2^/m^2^ for obese women [6]. Obesity is defined as a BMI of 30 or greater. Post-HIPEC CT scans are performed between 3 and 6 months after surgery to initiate surveillance, or as indicated to evaluate for complications. Patients with imaging between 2 and 4 months after HIPEC were evaluated for postoperative muscle mass.

We compared outcomes and demographic variables by sarcopenia status using Wilcoxon rank-sum tests for continuous variables and chi-squared tests for categorical variables. Post-HIPEC survival was defined as the number of days from the date of surgery to the date of death. Patients who were still alive at the time of analysis were considered censored. The method of Kaplan and Meier [7] was used to estimate survival curves by sarcopenia status, and the logrank test was used to test for differences between sarcopenia groups. Our protocol was approved by the Penn State Institutional Review Board, STUDY9023. Informed consent was waived for this study due to the retrospective nature of the study and the high mortality rate of the patient population.

## Results

**FIGURE 2:**
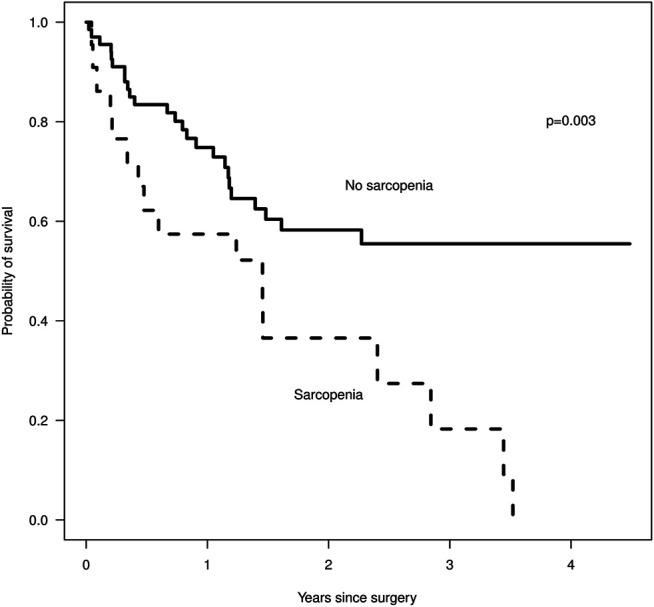
Survival after surgery by sarcopenia status, Kaplan-Meier, where *n* = 22 for the sarcopenic group and *n* = 67 for the non-sarcopenic group.

We identified 89 patients eligible for review and found that 25% had sarcopenia. Sarcopenia was associated with a higher PCI, median of 19 vs. 14 (*p* = 0.06) and shorter overall survival (Figure 2), median of 1.5 years vs. not reached by 4 years (*p* = 0.003), but sarcopenia did not predict a lower rate of complete cytoreduction, with 59 vs. 62% having a CC0/1 cytoreduction, *p* = 0.9. Our patients with sarcopenia had a statistically significantly lower BMI than the non-sarcopenic patients, 25.7 vs. 29.3, *p* = 0.002, yet their BMI was within normal range (Table 1). A majority of patients did not have weight loss prior to the scan. There was no difference in post-operative complication rate, age, gender or serum albumin level between sarcopenic and non-sarcopenic patients. Sarcopenic patients were more likely to have received chemotherapy, 77 vs. 58%, although this was not statistically significant, *p* = 0.11, and a longer length of stay, 10 vs. 8 days, although this was also not statistically significant (*p* = 0.12). The findings were similar when including the entire HIPEC population or selecting for appendiceal and colorectal cancer only. We identified 18 patients with CT scans between 2 and 4 months after HIPEC, with five of the 18 patients being sarcopenic prior to HIPEC. Post HIPEC muscle mass evaluation demonstrated that the non-sarcopenic patients remained non-sarcopenic. Only one of the sarcopenic patients remained sarcopenic, the other four patients had improved muscle mass and were within normal range. The four patients with sarcopenia resolution had an average post-HIPEC survival of 2.47 years (945 days) compared to the remaining sarcopenic patients, who had an average post-HIPEC survival of 1.09 years (401 days).

**TABLE 1:** Comparison of sarcopenic and non-sarcopenic patients.

	Sarcopenic *n* = 22	Non-sarcopenic *n* = 67	*p* Value
BMI, median (IQR)	25.7 (21.6–28.1)	29.3 (24.6–33.2)	0.002
PCI, median (IQR)	19 (11–26)	14 (6–24)	0.06
LOS, days, median (IQR)	10 (7–11)	8 (6–10)	0.12
Sys chemo*, *N* (%)	17 (77%)	39 (58%)	0.11
Age, median (IQR)	57 (20–77)	58 (25–82)	0.82
Sex, female, *N* (%)	11 (50%)	38 (57%)	0.58
Duration of disease^#^, median (IQR)	10.6 (7.3–20.3)	11.5 (2.6–28.3)	0.95
Albumin, median (IQR)	3.7 (3.1–4.3)	4.1 (3.4–4.4)	0.32
Primary Tumor:
Colon, *N* (%)	9 (41%)	26 (39%)	0.86
Appendix, *N* (%)	7 (32%)	22 (33%)	0.93
Lamn, *N* (%)	2 (9%)	3 (4%)	0.41
Ovary, *N* (%)	1 (5%)	5 (7%)	0.64
Other^, *N* (%)	3 (14%)	11 (16%)	0.76

*Any systemic chemotherapy during 1 year prior to scan. ^#^Time from diagnosis to CRS/HIPEC in months. ^∧^Gallbladder, gastric, desmoplastic small round cell, endometrium, rectum, mesothelioma, unknown primary, pancreas.

IQR, Interquartile range; BMI, body mass index; PCI, peritoneal cancer index; LOS, length of stay; LAMN, low grade mucinous neoplasm of appendix.

## Discussion

Sarcopenia is defined as low skeletal muscle mass and is conventionally measured on cross-sectional imaging at the level of the third lumbar vertebrae. Many factors can be associated with sarcopenia including age, malignancy, co-morbidities, gender and malnutrition [8, 9]. Sarcopenia has been associated with decreased survival time in patients with stage 1–3 colorectal cancer [6] and lower disease free and overall survival rates after resection of colorectal liver metastases [10]. Patients undergoing CRS/HIPEC for colorectal cancer metastases have an increased risk of severe postoperative complications [2] and increased toxicity from the intraperitoneal chemotherapy [1] if they are sarcopenic, making sarcopenia a relevant marker in this patient population for both short- and long-term poor outcomes.

Sarcopenia was present in 25% of our HIPEC patients and was an indication of a higher burden of disease and shorter survival time. The rate of sarcopenia was lower than described in other series [2, 6, 11], although patients who are candidates for CRS/HIPEC represent a highly select group of patients with metastatic disease. Nutritional parameters such as serum albumin level are often used to predict patient outcome [12] and the risk of complications, however in this patient population there was no difference between groups, and the sarcopenic patients had an albumin level considered within normal range. Similarly, the sarcopenic patients had a BMI within normal range, and no history of recent weight loss. These findings are consistent with other reports and have several important implications. The presence of sarcopenia may not be clinically apparent and all patients should have muscle mass calculated. The presence of sarcopenia has significant consequences for patient outcome and may help in counseling and selecting patients for surgery.

Patients who received systemic therapy prior to HIPEC were more likely to be sarcopenic, although this did not reach statistical significance likely due to the small number of patients in this study. With the wide variety of tumor types and timing of presentation for HIPEC there was significant variability in the chemotherapy regimens and duration of treatment. For this small number of patients we elected to separate them into “any chemotherapy” and “no chemotherapy” groups. Previous work has demonstrated that a high percentage of patients will have loss of skeletal muscle mass during chemotherapy treatment [13], and sarcopenia is associated with higher rates of treatment related complications and inability to complete prescribed chemotherapy regimens [14]. Strategies to maintain muscle mass can lead to improved tolerance of treatment [15] and reduced side effects [16]. A majority of patients who are candidates for CRS/HIPEC will be eligible for and receive systemic therapy at some point during their treatment. While the impact of muscle mass on treatment related complications is clear, it is unknown whether treatment planning based on muscle mass will improve outcome. For example, a patient with sarcopenia may have a better outcome with CRS/HIPEC first followed by systemic therapy rather than systemic therapy first.

The improvement in muscle mass after HIPEC was unexpected, particularly among the patients who already had sarcopenia. We anticipated further loss of muscle in the short term after the stress of a long operation and intraperitoneal chemotherapy. The number of patients is too small to draw statistically significant conclusions, but the finding suggests that elimination of the burden of cancer may improve a patient’s physiology. Alternatively, those patients who are physiologically able to build muscle mass after CRS/HIPEC may represent the select group of patients with better disease biology. In this study we chose to focus on short-term muscle mass and therefore did not evaluate scans more than 4 months after HIPEC. Additional longitudinal studies that measure muscle mass over the entire course of a patient’s treatment should be performed.

Sarcopenia may be an indicator of more advanced systemic disease, a longer duration of treatment or both. It is not well documented in the literature if sarcopenia can be reversed in this patient population, or if addressing sarcopenia can alter patient outcomes. Further studies are needed in order to better understand the effect of sarcopenia on this patient population, including interventional trials that attempt to reverse loss of muscle mass. Our study is limited in its retrospective nature as well as the small sample size. Additionally, not all patients received CT scans in the 2–4 months following their HIPEC procedure thus reducing our sample size further for post HIPEC sarcopenia rate. It is possible that this biased our results in finding that only 1/5 patients continued to have sarcopenia following HIPEC.

## Conclusion

Sarcopenia is present in patients with advanced malignancy and may be clinically occult. The presence of sarcopenia is associated with worse short and long-term outcomes including survival. Larger studies are needed in this patient population and interventional trials should be considered.

## Data Availability

The original contributions presented in the study are included in the article/Supplementary Material, further inquiries can be directed to the corresponding author.
